# Elizabeth Bagshaw: Pioneer of Canadian Women’s Health

**DOI:** 10.7759/cureus.64845

**Published:** 2024-07-18

**Authors:** Renish N Contractor, Kalani Dempsey, Manav Shah, Andrew Lazo, Prathap Simhadri

**Affiliations:** 1 Urology, Florida State University College of Medicine, Daytona Beach, USA; 2 Medicine, University of West Florida, Pensacola, USA; 3 Medicine, University of Florida, Gainesville, USA; 4 Internal Medicine, University of South Florida, Tampa, USA; 5 Internal Medicine/Nephrology, AdventHealth, Florida State University College of Medicine, Daytona Beach, USA

**Keywords:** historical vignette, women's health, birth control, elizabeth bagshaw, medical history

## Abstract

Dr. Elizabeth Bagshaw, an esteemed figure among Canada’s female physicians, devoted over seven decades to advancing obstetrics and reproductive health. She defied conventional norms by pursuing medical education and graduating from the Ontario Medical College for Women in 1905. Throughout her illustrious career, Bagshaw demonstrated exceptional perseverance, navigating familial obligations following her father’s untimely passing during her academic pursuits.

Establishing her practice in Hamilton, Ontario, Bagshaw delivered over 3,000 neonates, frequently offering pro bono care to immigrant populations. Notably in 1932, Bagshaw assumed the role of Medical Director of Canada’s first birth control clinic, challenging restrictive legislation and paving the way for the legalization of contraception in 1969. Bagshaw’s efforts provided women with vital reproductive health services and information, significantly impacting public attitudes and legislation.

Beyond her medical practice, Bagshaw also played a pivotal role in mitigating public health crises, including the Spanish flu, and ventured into politics with a city council campaign in 1934, supported by The Women’s Civic Club. Her extensive contributions earned her numerous accolades, including posthumous induction into the Canadian Medical Hall of Fame in 2007.

Bagshaw’s enduring legacy is reflected in the Elizabeth Bagshaw Clinic, which continues to offer reproductive and abortion care in a confidential and supportive setting. Bagshaw’s pioneering work significantly advances health equity and women’s reproductive rights, leaving a lasting impact on healthcare worldwide. Her life and achievements underscore her role as a tireless advocate for women’s health and a transformative influence in medical history.

## Introduction and background

Early life and education

Elizabeth Bagshaw, one of Canada’s first female physicians, was a radical advocate for women’s health over 70 fruitful years spent in obstetrics and reproductive health. Bagshaw was born on October 18, 1881, on a farm in Cannington, Ontario, to John and Eliza Bagshaw and was the youngest of four daughters. Elizabeth was set to break the mold, leaving her family farm in hopes of pursuing a career in medicine.

**Figure 1:**
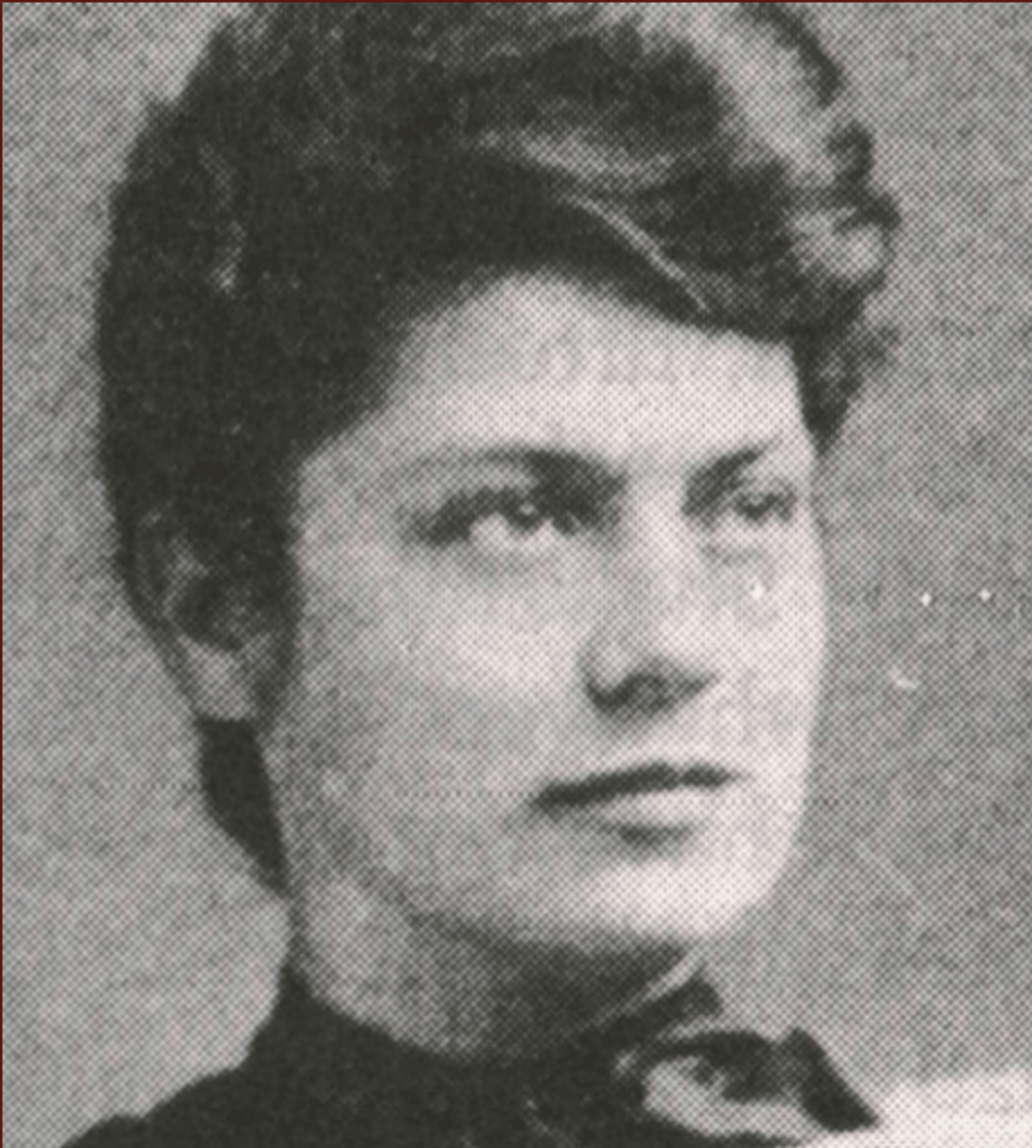
Portrait of a young Elizabeth Bagshaw Image Credit: Permission obtained from the Canadian Medical Hall of Fame

By 1901, she had enrolled at the University of Toronto at a mere 19 years old (Figure 1). Though mocked by her peers for not pursuing a career in the arts, Elizabeth was steadfast in her dedication to science and graduated in 1905 from Ontario Medical College for Women, which was affiliated with the University of Toronto, with a degree in medicine. This made her one of the first female doctors in Toronto [1].

## Review

Early career

Elizabeth was no stranger to the adversity she faced as a female physician in the early 20th century. Her resilience was tested early in medical school when her father’s sudden death in a farming accident in 1904 placed the responsibility of managing the family farm squarely on her shoulders. Despite this notable setback, Elizabeth efficiently and quickly managed the affairs of the farm. She promptly resumed her medical studies, taking on the additional responsibility of caring for her mother and sister. This event underscores Elizabeth’s remarkable adaptability and determination in navigating personal and professional obstacles during her career in medicine [2].

Following matriculation in 1905, Elizabeth quietly worked as an intern and resident under other male physicians, careful not to ruffle societal feathers. During this period, Bagshaw faced systemic segregation and discrimination. She endured taunts from her male peers in predominantly gender-segregated classes [3]. In the documentary “Doctor Woman” (1978), Bagshaw reflected on the limited opportunities for women in surgery; she notes “I’d [have] liked to have gone through for a surgeon, but in those days there was no chance. They wouldn’t have trusted a woman in those days to be a surgeon" [4]. This set the path for Bagshaw to enter family practice and focus on women’s health and obstetrics. 

Hamilton clinic

Soon thereafter, Elizabeth went on to open her practice in Hamilton, Ontario, where she spent 70 years practicing family medicine, specializing in reproductive health and obstetrics. Elizabeth cared for immigrant patients, who often felt more comfortable with a female physician due to their culture of midwifery. Many of her patients felt more comfortable having a female physician perform their obstetric and gynecological care. At this time, Bagshaw was the only female physician providing such services in the Hamilton community. Bagshaw often saw patients at no cost, where her heart of service showed proudly. This allowed patients who previously would not have received care access to healthcare services. This was especially prominent during the Great Depression, when there were no welfare and no unemployment payments. Delivering 3000 babies was an achievement in itself [5], but Elizabeth Bagshaw did not stop there. Her entry into a profession where women were previously not allowed helped normalize women’s presence in medicine. 

By 1932, she had become the Medical Director of Canada’s first birth control and family planning clinic. It was in operation despite an 1892 legislation, which outlawed family planning and abortion clinics. Those who were caught and convicted faced up to two years imprisonment. Despite this, Bagshaw’s work remained underground for decades as it was illegal to provide information on birth control practices or to provide patients with contraceptive methods. Elizabeth’s rebellious archetype earned her a legacy as she continued to operate and provide care for those in need [4].

Advocacy

Dr. Bagshaw remained in this position for over 30 years, empowering Hamilton women to control their reproductive health and family planning. In Hamilton, Bagshaw’s clinic was the only of its kind to provide these services for women. At this clinic, for the first time, Canadian women had a space to learn about their bodies and felt safe to express their reproductive choices. Bagshaw’s pioneering work helped to change attitudes and eventually the law. A 1934 study showed that one in six deaths among young women in the state of Ontario was due to pregnancy and childbirth. Illegal abortions provided by non-healthcare professionals also contributed to high maternal death rates. Bagshaw’s clinic helped women feel safer during childbirth and abortions and likely led to fewer deaths among young women. In 1969, three years after Bagshaw retired from the clinic, Canada reversed its law on birth control [4].

In addition to the strides made in women’s health, Elizabeth was a notable figure during the Spanish flu and Prohibition era and within the Hamilton community. She graciously tended to those in the community suffering from tuberculosis, smallpox, and typhoid. It is noted that Bagshaw herself contracted the Spanish flu [6], one of the largest pandemics in history. She fought fervently and beat the odds, which killed millions around the world. In 1934, Bagshaw expanded her pursuits to the political circle by running for city council. Affiliated with and supported by The Women’s Civic Club, Bagshaw attained a total of 2,278 votes in her campaign on December 3, 1984. Despite thousands of votes, Elizabeth finished third in the race, being succeeded by William MacFarland. Elizabeth was also a proud member of the Zonta Club of Hamilton from 1937 to 1982. This all-women’s club was organized in 1919 to promote education for girls and young women in Hamilton [7]. Elizabeth’s fierce independence, curiosity for science, and nurturing desire earned her various awards including Hamilton’s Citizen of the Year award (1970), the Honorary Doctor of Laws achievement (1974), and induction into Canada’s Medical Hall of Fame (2007). These awards only scratch the surface of the hallmarks Elizabeth left on medicine [8]. 

After an illustrious career, Dr. Bagshaw retired in 1976, at the age of 95. At the time, she was Canada’s oldest practicing physician [5]. Her 70-year career spanned many eras in medicine and law. Bagshaw’s clinical accomplishments helped change the lives of thousands of women in Hamilton. Bagshaw’s advocacy helped pave the way for future female physicians and changed the way we view women’s health and family planning. Her career and passion for women’s health paved the way for future female physicians in Canada, helping Canadian women live out their dream of becoming a physician.

Legacy and impact

Dr. Bagshaw’s legacy carries on through the Elizabeth Bagshaw Clinic, which was founded in 1990 in honor of her illustrious career. This non-profit accredited medical facility aims to provide reproductive and abortion care to women, trans, and non-binary people. It aims to carry on Dr. Bagshaw’s mission of providing care in a safe, confidential, and nonjudgmental atmosphere and supports clinical teaching and research through the University of British Columbia. The clinic serves as a haven for abortions and reproductive counseling in a patient-focused and supportive environment with respect for patient autonomy [9].

**Figure 2:**
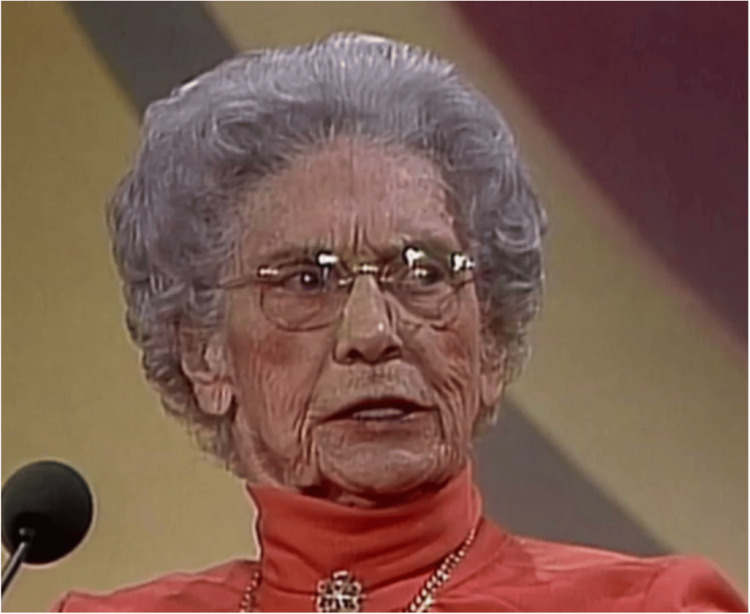
Dr. Bagshaw honored for Canadian women’s health advocacy Image Credit: Permission obtained from the Canadian Medical Hall of Fame

For her work as a medical pioneer, Bagshaw was posthumously inducted into the Canadian Medical Hall of Fame in October 2007 (Figure 2). Bagshaw’s courage in the face of scrutiny and prejudice led to her contributions to health equity for Canadian women. Working as a tireless champion for women’s reproductive rights, her impact can be seen across the world today, where women are seen as equals and their healthcare needs are recognized and attended to. Her contributions aided in the Morgentaler decision that led to the legalization of abortion in 1988 [1]. Today, there is more access to contraception than ever before, and this is partly due to the contributions of Elizabeth Bagshaw.

In 1978, the National Film Board of Canada released a film in Bagshaw’s honor named Doctor Woman: The Life and Times of Dr. Elizabeth Bagshaw. Written by Nancy C. Johnson and Kem Murch, this movie was aimed to retell the anecdote of Dr. Bagshaw’s struggles and accomplishments in Canadian medicine [8]. It focused on Bagshaw serving as a forerunner of the women’s movement, from being one of the first Canadian women in medicine to her advocacy. During her 70-year career, she helped lead the change in society’s perception on women’s rights and use of contraception.

Elizabeth was also honored by McMaster University’s In Her Hands: A Century of Women Shaping Healthcare in Hamilton, an exhibit presenting influential women in medicine in the town of Hamilton, Canada [10]. This showcase told of the various nurses, doctors, midwives, geneticists, and politicians; Elizabeth Bagshaw was in good company with many other powerful, bright women. The city of Hamilton continued to profess their adoration, selecting Dr. Bagshaw as the Citizen of the Year in 1970. 

Aptly fitting her hyper-independent nature, Elizabeth never married. However, it is believed Elizabeth held a spot in her heart for Lou Honey, having kept photos of him mounted to her wall after his passing in the war [1]. Before Lou died in 1915, he gave Elizabeth a diamond ring. Elizabeth would have been 34 at the time of his death, losing their dream to wed. In addition to Lou, Elizabeth adopted two children from a relative in her 40s, John and Voureen [11]. Though Elizabeth Bagshaw’s life drew to an end at age 100, her passion for helping women is duly noted both in and out of the clinic.

## Conclusions

It is evident that Elizabeth Bagshaw had a bountiful, fulfilling career as both a clinician and an advocate. Her compassion for her community, women, and education are just a few measures that earned her a legacy, even at a young age. Bagshaw prevailed despite numerous obstacles and surpassed all expectations. Establishing her practice in Hamilton, Ontario, Bagshaw delivered over 3,000 neonates, frequently offering pro bono care to immigrant populations. Notably in 1932, Bagshaw assumed the role of Medical Director of Canada’s first birth control clinic, challenging restrictive legislation and paving the way for the legalization of contraception in 1969. Bagshaw’s efforts provided women with vital reproductive health services and information, significantly impacting public attitudes and legislation. Dr. Elizabeth Bagshaw spent nearly 81 years practicing medicine and 100 years shaping history. Due to her efforts, access to birth control is greater than ever, and women today have more autonomy in their reproductive rights.
